# Behaviours that Challenge in SATB2-associated Syndrome: Correlates of Self-injury, Aggression and Property Destruction

**DOI:** 10.1007/s10803-023-06123-2

**Published:** 2023-09-26

**Authors:** Lauren Shelley, Jane Waite, Joanne Tarver, Chris Oliver, Hayley Crawford, Caroline Richards, Stacey Bissell

**Affiliations:** 1 College of Health and Life Sciences, Aston University, Birmingham, UK; 2 School of Psychology, University of Birmingham, Edgbaston, Birmingham, UK; 3 Mental Health and Wellbeing Unit, Warwick Medical School, University of Warwick, Coventry, UK; 4 Cerebra Network for Neurodevelopmental Disorders, University of Birmingham, Birmingham, UK

**Keywords:** SATB2-associated syndrome, SATB2, Self-injury, Aggression, Property destruction, Behaviours that challenge

## Abstract

SATB2-associated syndrome (SAS) is a genetic syndrome characterised by intellectual disability, severe speech delay, and palatal and dental problems. Behaviours that challenge (BtC) are reported frequently; however, there is limited research on specific forms of BtC and the correlates of these behaviours. The current study explores correlates of well-defined BtC, self-injury, aggression, and property destruction, in SAS. Eighty-one parents/caregivers of individuals with SAS (53.1% male, Mage 10.12 years) completed questionnaire measures of health, behavioural, emotional, and autism characteristics. Individuals with SAS were grouped based on caregiver responses to the presence or absence of self-injury, aggression, and property destruction on the Challenging Behaviour Questionnaire. Rates of self-injury, aggression and property destruction were 42%, 77% and 49%, respectively. Between-group comparisons were conducted to compare characteristics between behaviour groups. Significantly differing characteristics were entered into separate hierarchical logistic regressions for each form of BtC. Behavioural comparisons indicated variation in the characteristics associated with each behaviour. All hierarchical logistic regression models were significant (*p* < .001): self-injury (χ^2^(5) = 38.46, *R*^2^ = 0.571), aggression (χ^2^(4) = 25.12, *R*^2^ = 0.414), property destruction (χ^2^(4) = 23.70, *R*^2^ = 0.346), explaining between 34.6% and 57.1% of the variance in behaviour presence. This is the first study to identify correlates of self-injury, aggression, and property destruction in SAS. Variability in the characteristics associated with each behaviour highlights the importance of specificity when examining BtC. Understanding correlates of specific forms of BtC has important implications for informing SAS-associated pathways to behavioural outcomes and the implementation of tailored behavioural interventions.

SATB2-associated syndrome (SAS) is a rare genetic syndrome associated with variants causing functional haploinsufficiency of the *special AT-rich sequence-binding protein 2* gene (OMIM #608148) located on chromosome 2q33.1 (Zarate et al., 2019). SAS is estimated to occur in 0.2 to 0.3% of individuals with an undiagnosed intellectual disability or developmental delay (Bengani et al., 2017; Zarate & Fish, 2017). Clinical characteristics include moderate-to-profound intellectual disability, absent or delayed language acquisition, and craniofacial and dental abnormalities (Zarate & Fish, 2017; Zarate et al., 2018). To date, clinical characteristics are largely reported to be consistent across individuals, irrespective of variant (e.g., missense, nonsense, frameshift, intragenic deletion; Zarate et al., 2019).

Broadly categorised ‘behavioural issues’ are reported in 55% of individuals and are therefore considered a core feature of SAS (Zarate & Fish, 2017; Zarate et al., 2017). Behaviours of concern have predominantly been described through clinical case reports and retrospective review of clinical records. Few studies have used validated and standardised measures to investigate these behaviours. Although behavioural descriptions have varied across individuals, behaviours identified have included forms of behaviour directed towards others (e.g., agitation, scratching, hair pulling), behaviour towards oneself (e.g., skin picking, head banging), and behaviour directed towards objects (e.g., throwing objects), which may be categorised as aggression, self-injury and destructive behaviour, respectively (Balasubramanian et al., 2011; Van Buggenhout et al., 2005; Zarate & Fish, 2017; Zarate et al., 2018).

Aggression, self-injury, and destructive behaviour fall under the umbrella term ‘behaviours that challenge’ (BtC); defined as behaviours with an intensity, frequency, or duration as to impact the quality of life, physical safety, and/or emotional wellbeing of an individual or those around them (Emerson et al., 2001; Banks et al., 2007). In wider intellectual disability populations, BtC typically persist over time, and are associated with hospitalisation, poorer health, exclusion, and long-term use of psychotropic medication (Cooper et al., 2009; Davies and Oliver, 2016; Emerson et al., 2001; Taylor et al., 2011; Totsika and Hastings, 2009). Caregivers of individuals with SAS have anecdotally reported significant concern about these behaviours and their potential to persist and increase in severity. The persistence of BtC across the lifespan has been delineated cross-sectionally, revealing high rates of multiple forms of BtC in children, adolescents, and adults with SAS (Bissell et al., 2022). Further, a large proportion of caregivers have noted BtC are associated with distress in the person with SAS that increases with age (Cotton et al., 2020).

Despite the prevalence of BtC and the substantial impact reported by families, few research studies have focussed on the aetiology of these behaviours in SAS, including the person characteristics that may be associated with their occurrence. Examining these associations may allude to correlates for specific behaviours and inform hypotheses of mechanistic pathways underlying behaviour in SAS. Understanding these mechanistic pathways in SAS specifically is important, given research showing differences in profiles of BtC between and within syndrome groups (Arron et al., 2011), and differences in phenotypic mechanisms underpinning behavioural outcomes, such as differences in cognitive or anxiety characteristics.(Crawford et al., 2019; Cressey et al., 2019; Oliver, 2017; Wilde et al., 2018; Woodcock et al., 2009).

To thoroughly investigate pathways to behavioural outcomes in genetic syndromes it is important that BtC are defined well (Oliver, 2017). To date, most studies investigating BtC in SAS have lacked specificity in behaviour measurement, instead describing BtC at the broadest level of description (Cotton et al., 2020; Zarate & Fish, 2017; Zarate et al., 2018). In a study assessing the presence of specific categories of BtC in a large group of individuals with SAS, varying prevalence rates of behaviour were found; self-injury (43%), aggression (77%), and destructive behaviour (47%; Bissell et al., 2022). Despite varying rates of BtC being reported, it is currently unknown whether syndrome associated characteristics are associated with specific forms of BtC in SAS. A fine-grained approach to understanding different forms of BtC is important if research is to inform interventions to reduce these behaviours (Beavers et al., 2013). Particularly given research indicating gene-behaviour-environment interactions in the development of behaviour, and differing pathways to different forms of behaviour in some genetic syndromes (Davies & Oliver, 2016; Waite et al., 2014).

The presence of BtC has been associated with various syndrome-associated characteristics in the wider intellectual disability literature, and the presence of such characteristics in SAS may increase the likelihood of different forms of BtC. Moderate-to-profound intellectual disability is associated with increased prevalence of self-injury, aggression and destructive behaviour compared to mild intellectual disability (Cooper et al., 2009; McClintock et al., 2003). Delays in receptive and expressive communication are also associated with the occurrence and severity of BtC, such as self-injury (Holden & Gitlesen, 2006; Lundqvist, 2013; McClintock et al., 2003; Murphy et al., 2005; Oliver et al., 2012; Simó-Pinatella et al., 2017). Moderate-to-profound intellectual disability and delayed language and communication are core characteristics of SAS, which may increase the likelihood of BtC (Thomason et al., 2019; Zarate et al., 2021; Zarate & Fish, 2017).

Autism characteristics are more likely to occur in individuals with a genetic syndrome compared to the general population (Arron et al., 2011; Richards et al., 2015) and differing profiles of autism characteristics are indicated across syndromes, including those who meet or exceed cut-off on clinical screening tools (Moss et al., 2013). Autism characteristics, including differences in social interaction and communication, and restricted and repetitive patterns of behaviour, activities or interests, are associated with an increased likelihood of self-injury and aggression in individuals with intellectual disability (American Psychiatric Association, 2013;Arron et al., 2011; Duerden et al., 2012; Oliver et al., 2012; Richards et al., 2012). Autism characteristics are described in approximately 46% of individuals with SAS and current literature suggests a repetitive behaviour profile characterised by insistence on sameness, compulsivity, and stereotypy (Bissell et al., 2022; Zarate et al., 2019; Zarate & Fish, 2017).

In addition to repetitive behaviour, impulsivity and hyperactivity have been described in some individuals with SAS (Bissell et al., 2022; Zarate et al., 2020; Zarate & Fish, 2017). Increased levels of restricted and repetitive behaviour are frequently associated with increased levels of impulsivity, a behavioural marker of compromised behavioural inhibition that is robustly associated with the heightened prevalence and persistence of self-injury and aggression in autism and several genetic syndromes (Arron et al., 2011; Crawford et al., 2019; Laverty et al., 2020; Perry et al., 2022; Richards et al., 2016; Wilde et al., 2018). The behavioural dysregulation model proposes that the combination of impulsivity and restricted and repetitive behaviour influences the development and maintenance of self-injury through reduced executive function abilities that underpin compromised behavioural self-regulation (Oliver & Richards, 2015). The combined presence of high levels of repetitive behaviour and impulsivity in some individuals with SAS alludes to a role for compromised behavioural regulation that might be important for understanding the development and maintenance of BtC in this group.

Health difficulties, pain, and behavioural indicators of pain are also robustly associated with increased frequency and intensity of BtC in individuals with intellectual disability (Carr & Owen-DeSchryver, 2007; Hastings et al., 2013; Richards et al., 2016). In SAS, physical health issues including dental problems, gastrointestinal difficulties, recurrent ear infections, scoliosis and osteoporosis, may increase the likelihood of pain and discomfort and contribute to the presence or severity of BtC (Kumar & Zarate, 2020; Scott et al., 2019; Zarate et al., 2018, 2021). Pain and discomfort are additionally associated with distress and anxiety symptomology in individuals with intellectual disability (Hayes et al., 2011; Oliver et al., 2012; Oliver and Richards, 2015). Anxiety symptomology is prevalent in intellectual disability populations (Edwards et al., 2022a; Mazza et al., 2020), and behavioural indicators of anxiety include BtC (Edwards et al., 2022b; Moskowitz et al., 2013; Painter et al., 2018; Tarver et al., 2021). A susceptibility to internalising problems, including anxiety, is reported in individuals with SAS, suggesting anxiety might contribute to BtC in this group (Cotton et al., 2020; Kumar & Zarate, 2020; Zarate et al., 2021).

In summary, health, behavioural, autism and emotional characteristics associated with SAS suggest an increased likelihood for the presence of BtC, such as self-injury, aggression and property destruction. Although research in other genetic syndrome groups has demonstrated an association between several person characteristics and forms of BtC, these putative associations are yet to be evaluated in individuals with SAS. While characteristics associated with SAS may increase the likelihood for the occurrence of different forms of BtC, pathways to behavioural outcomes and the pattern of correlates for forms of BtC in SAS may be different compared to other syndrome groups. Ascertaining the characteristics that may be associated with specific categories of BtC in SAS is of significant value to help identify potential correlates of BtC in this group, and to inform SAS-associated models of behaviours which may have important implications for early intervention. To address the identified gaps in the literature, the current cross-sectional study aimed to:

1. Conduct between-group comparisons to examine differences in person characteristics (demographic, health, behavioural, autism and emotional characteristics) between individuals with SAS who show and do not show different categories of BtC (*self-injury, aggression, property destruction*).
2. Conduct multiple logistic regression analyses to identify person characteristics that may be potential correlates of risk for the presence of different categories of BtC in individuals with SAS.

## Methods

The current cross-sectional questionnaire study utilised between-group comparative analyses to examine differences in characteristics between individuals who do and do not show self-injury, aggression, and property destruction, and multiple logistic regression analyses to identify correlates associated with an increased likelihood of each form of behaviour. This approach enabled collection of a large dataset to capture characteristics associated with BtC and enable initial causal modelling of characteristics that might give rise to specific forms of BtC.

### Participants

The current study is a secondary analysis of an existing dataset held by the Cerebra Network for Neurodevelopmental Disorders. Participants were caregivers of 81 individuals with SAS (mean age: 10.12 years (age range: 1–36 years; 53.1% male), first recruited via SAS family support group organisations (see Bissell et al., 2022). Inclusion criteria required individuals with SAS to be aged 1 year or above with a clinical diagnosis of SAS. All individuals had received a clinical diagnosis from a general practitioner, paediatrician, clinical geneticist, or neurologist. Where caregivers consented to genetic confirmation sharing and records were available, diagnosis was further confirmed for 33 individuals (40.7%) via sharing of clinical genetic confirmation letters. Ethical approval for the original study was granted by Coventry and Warwickshire NHS Research Ethics Committee (reference: 10/H1210/1).

### Procedure

Caregivers of children and adults with SAS accessed and completed an information sheet, consent forms and questionnaire measures (see *Measures*), via an online survey hosted with LimeSurvey 2.00 + software (GmbH, 2012).

### Measures

Full descriptions of the measures used and their psychometric properties are presented in Table 1. The measures selected for this study have been used in previous studies with people with genetic syndromes. Further, many measures were considered appropriate for use in the current study due to being designed for use with people with moderate to profound intellectual disability.

**Table 1:** Description of questionnaire measures

Measure	Description
Background Questionnaire	A background questionnaire collected demographic information about the participant with SAS, including age, sex, verbal ability, mobility, and diagnostic information (e.g., professional from whom the diagnosis was received and date of diagnosis).
Wessex Questionnaire (WQ; Kushlick et al., 1973)	Proxy measure of adaptive ability in individuals with intellectual disability. The 16-item questionnaire asks about vision, mobility, speech, hearing, literacy, continence, and self-help. Items are rated on a three-point scale ranging from 1 (severe impairment) to 3 (no impairment). Items measuring a person’s ability to independently wash, dress and feed are added to form an overall self-help score, ranging from 3 (not able) to 9 (able). The authors of the questionnaire report good inter-rater reliability, with agreement percentages ranging from 78–92% (Kushlick et al., 1973).
Social Communication Questionnaire – Lifetime Version (SCQ-L; (Berument et al., 1999)	Provides an indication of characteristics of autism spectrum disorder. Informants rate a total of 40 dichotomous items with response options of yes or no. Responses are grouped into subscales measuring reciprocal social interaction, communication difficulties, and restricted, repetitive, and stereotyped behaviour. Higher scores indicate a higher number of autism characteristics, with scores of ≥ 15 indicating autism spectrum condition and scores of ≥ 22 indicating autism. The SCQ-L has excellent internal consistency (α = 0.90), good concurrent validity with both the Autism Diagnosis Interview and the Autism Diagnostic Observation Schedule and is suitable for individuals with intellectual disability aged 4 years and over (Berument et al., 1999).
Health Questionnaire (HQ; Hall et al., 2008)	Examines the presence and severity of current (in the past month) health problems. Informants score a total of 15 health problems on a four-point scale from 0 (never affected) to 3 (severely affected). Scores were summed to obtain current health frequency and severity scores. Hall et al. (2008) report good item-level reliability for current health problems (0.76).
Gastro-oesophageal Distress Questionnaire (GDQ; Oliver & Wilkie, 2005)	Consists of 17 questions to assess behaviours that are indicative of gastro-oesophageal reflux. For the first 12 questions, informants rate the frequency of behaviours observed in the previous two weeks (e.g., regurgitate, cough, gag) on a five-point scale from 0 (not occurred) to 4 (more than once an hour). The remaining 5 questions assess lifetime behaviours and are rated with a combination of yes/no response options and a four-point scale from 0 (never) to 3 (every night). A score of ≥ 2 or response of yes is indicative of a cut-off for each item. The total score is calculated from the total number of items reaching cut-off. A total score of ≥ 5 is indicative of reflux.
Repetitive Behaviour Questionnaire (RBQ; Moss & Oliver, 2008)	Informant questionnaire used to record the occurrence of repetitive behaviours. Informants rate the frequency of 19 operationally defined behaviours on a five-point scale ranging from 0 (never) to 4 (more than once a day). The measure consists of five subscales of repetitive behaviour (insistence on sameness, compulsive behaviour, stereotyped behaviour, restricted preferences, and repetitive speech). The repetitive speech and restricted preferences subscales are not calculated for individuals who are minimally verbal. A higher score indicates a greater frequency of repetitive behaviour. The RBQ has good concurrent validity with the restrictive, repetitive, and stereotyped behaviour subscale of the SCQ-L (0.60), good internal consistency (0.80) at full-scale level, and acceptable to good inter-rater reliability (0.46 to 0.80 at item level with 73% of items above 0.60).
The Activity Questionnaire (TAQ; Burbidge & Oliver, 2008)	Informant-report measure of behavioural overactivity, impulsivity, and impulsive speech. Informants rate the frequency of 18 items on a five-point scale ranging from 0 (never/almost never) to 4 (always/almost all of the time). The measure consists of three subscales: overactivity, impulsivity, and impulsive speech. Higher scores on each subscale indicate greater behavioural severity. The impulsive speech subscale is not calculated for participants who are non-verbal. The TAQ has good inter-rater reliability and test-retest reliability at both total and subscale score level (≥ 0.70).
Challenging Behaviour Questionnaire (CBQ; Hyman et al., 2002)	An informant-report measure to assess the presence of behaviours that challenge within the past month on a yes/no basis. Behaviours include self-injury, physical aggression, property destruction and stereotyped behaviour. A self-injury score can be calculated out of 14 based on the reported frequency, severity, and frequency, where higher scores indicate higher levels of self-injury severity. As items assessing frequency, severity and frequency were only available for self-injury, the current study adopted a consistent approach and assessed the presence or absence of each behaviour on a dichotomous yes/no basis. Presence of stereotyped behaviour from the CBQ is not reported in the current study as a more detailed description of stereotyped behaviour is available from the SCQ. The CBQ has good concurrent validity with the Aberrant Behavior Checklist (0.56), and moderate to very strong inter-rater reliability (0.60-0.92).
Anxiety Depression and Mood Scale (ADAMS; Esbensen et al., 2003)	Informant-report measure of observable symptoms relating anxiety, depression, and mood. The questionnaire was originally developed for individuals with mild to profound intellectual disability. Informants rate a total of 28-items on a four-point scale ranging from 0 (not a problem) to 3 (severe problem). The questionnaire consists of five subscales (manic/hyperactive behaviour, depressed mood, general anxiety, social avoidance, and compulsive behaviour). Higher scores indicate a higher severity of symptoms. A test-retest reliability of 0.81 and internal consistency of 0.80 has been reported.
Mood Interest and Pleasure Questionnaire – Short Form (MIPQ-S; Ross, Oliver & Arron, 2008)	Informant-report measure of affect that is appropriate for use in individuals with intellectual disability. The measure consists of 6-items measuring mood in the previous two weeks and 6-items measuring interest and pleasure in the previous two weeks. Each item is rated on a five-point scale from 0 (never) to 4 (all of the time). Higher scores on each subscale indicate a more positive mood and higher interest and pleasure. The MIPQ-S has good test-retest reliability (0.97), inter-rater reliability (0.85), and good internal consistency for both the subscales and total score (≥ 0.79).

### Data Analysis

Data were analysed using Statistical Package for Social Sciences (SPSS), version 25.0. All data were tested for normality and homogeneity of variance (HOV) using Shapiro-Wilks and Levene’s tests, respectively. Distribution value and standard error (SE) statistics greater than 1 *SD* (1.96) were considered to violate skewness and kurtosis.

### Between-group Behavioural Comparisons

To conduct behavioural comparisons, participants were divided into groups. Group membership was based on caregiver-responses on the Challenging Behaviour Questionnaire (CBQ; see Table 1), indicating the presence or absence of different categories of BtC (*self-injury, aggression* and *property destruction*). Chi-square and Fisher’s exact tests were conducted to compare categorical demographic characteristics between behavioural groups. Parametric (Independent *t*-test)^1^ and non-parametric (Mann-Whitney *U*) tests were conducted to compare continuous person characteristics between behavioural groups; tests were chosen on a comparison-by-comparison basis, according to the normality and HOV of data.

### Hierarchical Logistic Regression Analyses

Separate hierarchical logistic regressions were conducted to identify predictors of binary outcome variables (0 = behaviour absent; 1 = behaviour present): *self-injury, aggression*, and *property destruction*. Predictors entered in each logistic regression were those shown to differ significantly in the relevant behavioural comparison analyses. Self-injury behavioural comparisons highlighted a number of potential predictors. Therefore, to minimise the number of predictor variables entered in the self-injury regression, and adhere to suggested rules of events per variable for logistic regression analyses, significant subscales within a measure were combined to form composite scores (Peduzzi et al., 1996; Vittinghoff & McCulloch, 2007). Hierarchical regression analyses were chosen to enable inputting of demographic and pathognomonic physical health characteristics, such as long-term dental problems, earlier in the model. After controlling for these characteristics, later steps included characteristics associated with BtC in the wider intellectual disability literature. This enabled examination of the additional contribution of autism, behavioural and emotional characteristics in the prediction of each behavioural outcome after controlling for demographic and pathognomonic health characteristics.

Data checks were carried out in each analysis to ensure the data did not violate assumptions for binary logistic regression. The Box-Tidwell procedure (Box & Tidwell, 1962) was used to assess linearity.^2^ Correlation coefficients (CC) and variation inflation factor (VIF) values were inspected to examine multicollinearity, which identified minimal autocorrelation (*self-injury* CC: 0.13 to 0.68, VIF: 1.48 to 2.70; *aggression* CC: -0.11 to -0.65, VIF: 1.05 to 2.42; *property destruction* CC: 0.07 to 0.48, VIF: 1.07 to 1.45). Casewise diagnostics, Cook’s distance, leverage and dfBeta’s were used to assess for outliers, high leverage points and highly influential points. Cases with standardised residuals > ± 2.5 were classed as outliers. Outliers with high leverage (> 0.5) and/or influence (Cook’s distance or dfBeta ≥ 1) were considered to exert large impact on model parameters and excluded from analyses (Field, 2018; Stevens, 2002). This led to the removal of one outlier from the self-injury regression analysis; results presented for this regression are without the outlier.

Across all analyses alpha was adjusted to *p* < .01 due to multiple comparisons and to reduce the likelihood of Type I errors. This was chosen over a more stringent Bonferroni correction due to the focus on a rare and under-researched syndrome group and the exploratory nature of the study, where it was considered important to identify possible clinically relevant associations and correlates that should be explored in future research.

## Results

### Between-group Behavioural Comparisons

Behavioural comparisons were conducted to address the first aim, to examine group differences in person characteristics between individuals who did and did not show different categories of BtC. Comparisons of demographic characteristics are displayed in Table 2. See Supplementary Information Table 1 for all comparisons of health, behavioural, autism and emotional characteristics. Overall, self-injury, aggression and property destruction were reported in 43%, 77% and 47% of individuals, respectively. A singular form of BtC was reported in 27.2% (22) of individuals, two forms of BtC were reported in 28.4% (23) of individuals, and all three forms of BtC were reported in 27.2% (22) of individuals. Of those who showed two forms of BtC, aggression and property destruction were more frequently reported together than self-injury and aggression or self-injury and property destruction.

**Table 2:** Demographic characteristics of yes/no groups across categories of behaviours that challenge and associated comparative analyses

	Self-injury		Comparative analyses		Aggression		Comparative analyses		Property Destruction		Comparative analyses	
	Yes (n = 34)	No (n = 47)	Statistic	*p* value	Yes (n = 61)	No (n = 18)	Statistic	*p* value	Yes (n = 39)	No (n = 40)	Statistic	*p* value
Age (years) ^a, c^; median (IQR) or mean (*SD*)	8.10 (5.26–5.29)	7.32 (5.03–13.71)	764.00	.738	8.10 (5.31–14.92)	5.90 (3.08–11.45)	393.00	.068	8.71 (6.05–17.34)	5.56 (4.6-11.48)	509.00	**.008**
Wessex self-help score ^a, d^; median (IQR) or mean (*SD*)	5.00 (4.00-6.25)	6.00 (5.00–7.00)	563.00	.020	6.00 (4.50-7.00)	5.50 (4.75-7.00)	497.50	.536	6.00 (4.00–7.00)	6.00 (5.00–7.00)	714.00	.506
Sex ^b, c^; n (% male)	21 (61.8)	22 (46.8)	1.77	.188	34 (55.7)	7 (38.9)	1.58	.209	27 (69.2)	14 (35.0)	9.27	**.002**
Mobility ^b, d^; n (% fully mobile)	29 (85.3)	44 (93.6)	-	.270	57 (93.4)	15 (83.3)	-	.191	37 (94.9)	35 (87.5)	-	.432
Vision ^b, d^; n (% normal)	28 (82.4)	39 (83.0)	0.01	.941	51 (83.6)	15 (83.3)	-	1.00	36 (92.3)	30 (75.0)	4.30	.038
Hearing ^b, d^; n (% normal)	31 (91.2)	46 (97.9)	-	.304	58 (95.1)	17 (94.4)	-	1.00	38 (97.4)	37 (92.5)	-	.615
Speech ^b, d^; n (% verbal)	9 (26.5)	17 (36.2)	4.08	.356	21 (34.4)	3 (16.7)	2.07	.150	9 (23.1)	15 (37.5)	1.94	.163

*Notes.* Significant group differences (*p* < .01) highlighted in **bold**. a = Mann Whitney *U* statistic or *t-*test statistic for group comparison; median (IQR; Interquartile Range) reported where Mann Whitney *U* tests were conducted, mean (*SD*) reported where *t*-tests were conducted. b = Chi-square test statistic for group comparison. Where test statistic is not reported, there were less than five expected values in cells and Fisher’s exact test was performed. c = data derived from Background Questionnaire. d = data derived from Wessex Behavior Scale. e = *t*-test with logarithmic transformation, descriptive values derived from untransformed raw data for greater representativeness.

#### Self-injury

SAS individuals who showed *self-injury* (SAS + SIB) were reported to experience significantly more current health problems (*U*(1) = 449.50, *Z* = -3.413, *p* = .001, *r* = .38), greater severity of current health problems (*U*(1) = 437.50, *Z* = -3.508, *p* < .001, *r* = .39), and higher levels of behaviour indicative of gastro-oesophageal reflux (GDQ clinical signs; *U*(1) = 339.00, *Z* = -4.427, *p* < .001, *r* = .49), than those who did not show *self-injury* (SAS-SIB). Compared to SAS-SIB individuals, SAS + SIB individuals were also reported to show significantly higher levels of *impulsivity* (TAQ; *U*(1) = 465.00, *Z* = -3.207, *p* = .001, *r* = .36), *overactivity* (TAQ; *U*(1) = 387.00, *Z* = -3.945, *p* < .001, *r* = .44), *stereotyped behaviour* (RBQ; *U*(1) = 436.00, *Z* = -3.522, *p* < .001, *r* = .39), *restricted, repetitive and stereotyped behaviour* (SCQ; *U*(1) = 331.50, *Z* = -3.276, *p* = .001, *r* = .39), *reciprocal social interaction* difficulties (SCQ; *U*(1) = 372.50, *Z* = -2.785, *p* = .005, *r* = .33), *manic/hyperactive behaviour* (ADAMS; *U*(1) = 438.50, *Z* = -3.463, *p* = .001, *r* = .38), *general anxiety* (ADAMS; *U*(1) = 418.50, *Z* = -3.654, *p* < .001, *r* = .41), and *compulsive behaviour* (ADAMS; *U*(1) = 451.50, *Z* = -3.360, *p* = .001, *r* = .37).

#### Aggression

Compared to SAS individuals who did not show *aggression* (SAS-AGG), SAS individuals who showed *aggression* (SAS + AGG) were reported to show significantly higher levels of *impulsivity* (TAQ; *U*(1) = 259.50, *Z* = -3.395, *p* = .001, *r* = .38), *compulsive behaviour* (RBQ; *U*(1) = 317.00, *Z* = -2.724, *p* = .006, *r* = .31), *manic/hyperactive behaviour* (ADAMS; *U*(1) = 220.50, *Z* = -3.853, *p* = < 0.001, *r* = .43), and *general anxiety* (ADAMS; *U*(1) = 237.50, *Z* = -3.654, *p* = < 0.001, *r* = .41).

#### Property Destruction

Presence of *property destruction* (SAS + PD) was significantly associated with a higher chronological age (*U*(1) = 509.00, *Z* = -2.632, *p* = .008, *r* = .30). A significantly higher proportion of SAS + PD individuals were male (χ^2^(1) = 9.269, *p* = .002, *phi* = 0.34) compared to individuals who did not show *property destruction* (SAS-PD). SAS + PD individuals were also reported to show significantly higher levels of *compulsive behaviour* (RBQ; *U*(1) = 511.00, *Z* = -2.650, *p* = .008, *r* = .30) and *general anxiety* (ADAMS; *U*(1) = 384.00, *Z* = -3.897, *p* = < 0.001, *r* = .44), than SAS-PD individuals.

### Logistic Regression Analyses

Separate hierarchical logistic regression analyses were conducted to address the second aim, to ascertain the effect of predictor variables on the presence of *self-injury*, *aggression*, and *property destruction*.

#### Self-injury

Hierarchical logistic regression models for *self-injury* are presented in Table 3. At each step, the addition of each predictor variable led to a significant model compared to the constant-only model (*p* < .001). However, changes in log-likelihood ratios between each model did not reach significance (*p* > .01). There were no individual significant predictors of *self-injury* in the full hierarchical model, however, the predictors together distinguished between presence and absence of *self-injury* (χ^2^(5) = 38.46, *p* < .001). Overall, the full hierarchical model explained 57.1% (Nagelkerke *R*^2^) of the variance in presence of *self-injury* and correctly classified 77.1% of cases. Odds ratios indicate increasing clinical signs of gastro-oesophageal reflux were associated with increased likelihood of *self-injury*. While all other predictor variables were associated with increased likelihood of self-injury, 95% CIs ranged from < 1 to > 1, limiting confidence in whether each predictor led to an increase in the probability of *self-injury*.

**Table 3:** Hierarchical logistic regression analyses of presence of self-injury as a function of predictors

		CBQ self-injury									
Model and predictors		B	SE	Wald	Odds Ratio (95% CI)	Model *χ*^2^	df	− 2LL	Nag *R*^2^	∆ − 2LL χ^2^	*p*
Step 1						27.72	2	66.50	.442	27.72**	< .001**
	Health composite	.18	.15	1.41	1.19 (0.89–1.59)						.235
	GDQ score	.47	.14	11.39	1.59 (1.22–2.09)						.001**
	Constant	-3.37	.79	18.12	.04						.001**
Step 2						30.65	3	63.57	.479	2.93	< .001**
	Health composite	.20	.16	1.62	1.23 (0.90–1.68)						.203
	GDQ score	.36	.15	6.05	1.43 (1.08–1.90)						*.014*
	RBQ stereotyped behaviour	.14	.08	2.85	1.15 (0.98–1.35)						.092
	Constant	-3.64	.83	19.17	.03						.001**
Step 3						36.40	4	57.83	.548	5.74	< .001**
	Health composite	.25	.17	2.23	1.28 (0.93–1.78)						.135
	GDQ score	.38	.15	6.45	1.46 (1.09–1.96)						*.011*
	RBQ stereotyped behaviour	.02	.10	.03	1.02 (0.83–1.24)						.864
	SCQ composite	.41	.19	4.99	1.51 (1.05–2.17)						.025
	Constant	-5.33	1.27	17.79	.01						.001**
Step 4						38.35	5	55.87	.570	1.95	< .001**
	Health composite	.19	.18	1.18	1.21 (0.86–1.72)						.277
	GDQ score	.34	.15	5.19	1.41 (1.05–1.89)						.023
	RBQ stereotyped behaviour	− .01	.10	.00	1.00 (0.82–1.22)						.960
	SCQ composite	.43	.19	5.16	1.54 (1.06–2.24)						.023
	TAQ composite	.09	.06	1.85	1.09 (0.96–1.23)						.174
	Constant	-6.49	1.66	15.32	.00						
Step 5						38.46	6	55.77	.571	0.11	< .001**
	Health composite	.17	.19	.79	1.19 (0.82–1.73)						.373
	GDQ score	.33	.15	4.80	1.39 (1.04–1.88)						.028
	RBQ stereotyped behaviour	− .01	.10	.01	0.99 (0.81–1.22)						.941
	SCQ composite	.40	.21	3.64	1.50 (0.99–2.27)						.057
	TAQ composite	.07	.08	.90	1.07 (0.93–1.24)						.344
	ADAMS composite	.07	.20	.11	1.07 (0.72–1.59)						.746
	Constant	-6.39	1.67	14.64	.00						.001**

Note. N = 70. – 2 LL = − 2 Log Likelihood. Nag R2 = Nagelkerke R2. ∆ − 2LL χ2 values are the differences between – 2 LL by adding variables in each step to the model. Health composite = current health problem frequency and current health problem severity. TAQ composite = TAQ impulsivity and TAQ overactivity. SCQ composite = SCQ restricted, repetitive, and stereotyped behaviour and SCQ reciprocal social interaction. ADAMS composite = ADAMS manic/hyperactive behaviour, ADAMS general anxiety and ADAMS compulsive behaviour. Values approaching statistical significance highlighted in italics (deemed to trend towards significance if p = .011-0.014). * *p* < .01. **p < .001

#### Aggression

Hierarchical logistic regression models for *aggression* are presented in Table 4. The overall models at each step of the regression were significant. There were no individual significant predictors of *aggression* in the full hierarchical model, however, the predictors collectively distinguished between presence and absence of *aggression* (χ^2^(4) = 25.12, *p* < .001). Overall, the full hierarchical model explained 41.4% (Nagelkerke *R*^*2*^) of the variance in *aggression* and correctly classified 83.5% of cases. Odds ratios indicate that increasing levels of each predictor variable were associated with an increased likelihood of *aggression*; however, 95% CIs ranged from < 1 to > 1, limiting confidence in whether predictors led to increases in the probability of *aggression*.

**Table 4:** Hierarchical logistic regression analyses of presence of aggression as a function of predictors

		CBQ aggression									
Model and predictors		B	SE	Wald	Odds Ratio (95% CI)	Model *χ*^2^	df	− 2LL	Nag *R*^2^	∆ − 2LL χ^2^	p
Step 1						7.55	1	77.24	0.138	7.55*	0.006*
	RBQ compulsive behaviour ^a^	-1.77	0.64	7.58	0.17 (0.05–0.60)						0.006*
	Constant	1.72	0.36	23.43	5.62						< 0.001**
Step 2						18.76	2	66.04	0.321	11.21**	< 0.001**
	RBQ compulsive behaviour ^a^	-1.77	0.72	6.04	0.17 (0.04–0.70)						*0.014*
	TAQ impulsivity	0.17	0.05	9.53	1.18 (1.06–1.31)						0.002*
	Constant	− 0.90	0.86	1.10	0.41						0.294
Step 3						24.38	3	60.42	0.403	5.62	< 0.001**
	RBQ compulsive behaviour ^a^	-1.55	0.76	4.12	0.21 (0.05–0.95)						0.042
	TAQ impulsivity	0.08	0.06	1.46	1.08 (0.95–1.23)						0.228
	ADAMS manic/hyperactive behaviour	0.26	0.12	4.94	1.29 (1.03–1.62)						0.026
	Constant	-1.23	0.90	1.86	0.29						0.172
Step 4						25.12	4	59.68	0.414	0.74	< 0.001**
	RBQ compulsive behaviour ^a^	-1.46	0.79	3.45	0.23 (0.05–1.08)						0.063
	TAQ impulsivity	0.08	0.07	1.44	1.08 (0.95–1.23)						0.230
	ADAMS manic/hyperactive behaviour	0.19	0.14	1.97	1.21 (0.93–1.57)						0.161
	ADAMS general anxiety	0.10	0.12	0.68	1.11 (0.87–1.40)						0.410
	Constant	-1.26	0.93	1.84	0.29						0.175

*Note.* N = 79. – 2 LL = − 2 Log Likelihood. Nag *R*^2^ = Nagelkerke *R*^2^. ∆ − 2LL *χ*^2^ values (*df* = 1) are the differences between – 2 LL by adding each variable to the model. Values approaching statistical significance highlighted in italics (deemed to trend towards significance if p = .011-0.014). a = inverse square root transformation applied. *p < .01. **p < .001

#### Property Destruction

Hierarchical logistic regression models for *property destruction* are presented in Table 5. At each step, the addition of each predictor variable led to a significant model compared to the constant-only model (*p* < .001). There were no individual significant predictors of *property destruction* in the full hierarchical model, however, the predictors collectively distinguished between presence and absence of *property destruction* (χ^2^(4) = 23.70, *p* < .001). Overall, the full hierarchical model explained 34.6% (Nagelkerke *R*^2^) of the variance in *property destruction* and correctly classified 70.9% of cases. Odds ratios indicate that increasing chronological age and *compulsive behaviour* were associated with an increased likelihood of *property destruction*, while female sex was associated with a reduced likelihood of showing *property destruction*. Although *general anxiety* was associated with an increased likelihood of *property destruction*, 95% CIs ranged from < 1 to > 1, limiting confidence in whether *general anxiety* led to an increase in the probability of *property destruction*.

**Table 5:** Hierarchical logistic regression analyses of presence of property destruction as a function of predictors

		CBQ property destruction									
Model and predictors		B	SE	Wald	Odds Ratio (95% CI)	Model *χ*^2^	Df	− 2LL	Nag *R*^2^	∆ − 2LL χ^2^	p
Step 1						13.38	2	96.12	0.208	13.38**	0.001**
	Age	0.06	0.03	3.51	1.07 (1.00-1.14)						0.061
	Sex	-1.34	0.49	7.35	0.26 (0.10–0.69)						0.007*
	Constant	− 0.04	0.48	0.01	0.96						0.937
Step 2						22.26	3	87.25	0.327	8.87*	< 0.001**
	Age	0.07	0.04	4.13	1.08 (1.00-1.15)						0.042
	Sex	-1.67	0.56	9.10	0.19 (0.06–0.56)						0.003*
	RBQ compulsive behaviour	0.11	0.04	7.57	1.12 (1.03–1.22)						0.006*
	Constant	− 0.81	0.57	2.08	0.44						0.150
Step 3						23.70	4	85.81	0.346	1.44	< 0.001**
	Age	0.07	0.04	3.29	1.07 (1.00-1.15)						0.070
	Sex	-1.37	0.61	4.99	0.25 (0.08–0.85)						0.025
	RBQ compulsive behaviour	0.10	0.04	5.57	1.11 (1.02–1.21)						0.018
	ADAMS general anxiety	0.08	0.07	1.38	1.08 (0.95–1.23)						0.240
	Constant	-1.23	0.68	3.33	0.29						0.068

*Note.* N = 79. – 2 LL = – 2 Log Likelihood. Nag *R*^2^ = Nagelkerke *R*^2^. ∆ − 2LL *χ*^2^ values (*df* = 1) are the differences between – 2 LL by adding each variable to the model

*p < .01. **p < .001

## Discussion

This is the first known study to examine characteristics associated with the presence of self-injury, aggression, and property destruction in individuals with SAS. Findings revealed variability in the characteristics associated with the presence of each behaviour, demonstrating the importance of specificity to investigate well-defined categories of BtC. In the regression models for self-injury, aggression and property destruction, the Nagelkerke *R*^2^, which provides an effect size for logistic regression with a maximum value of 1 (Nagelkerke, 1991), increased between the null and full model, indicating that the addition of each predictor variable increased the proportion of variance explained by the models. Overall hierarchical regression models were statistically significant, each explaining a large proportion of variance in the presence of self-injury, aggression, and property destruction.

The findings in this study are consistent with previous research in genetic syndromes and intellectual disabilities. Health difficulties associated with pain and discomfort (Carr & Owen-DeSchryver, 2007), autism characteristics (Arron et al., 2011; Oliver et al., 2012; Richards et al., 2012) and impulsivity (Arron et al., 2011; Davies & Oliver, 2016) are commonly associated with self-injury. In the current study, comparative behavioural analyses revealed self-injury was more likely to be present in individuals with increased autism characteristics and higher levels of health difficulties, overactivity, and impulsivity. Presence of self-injury was also associated with higher levels of obsessive-compulsive, manic/hyperactive, and general anxiety characteristics. Regression models indicated variables explaining the most variance in the presence of self-injury were behavioural indicators of gastro-oesophageal reflux and socio-communicative differences. Although access to diagnostic records was not possible as part of this large-scale questionnaire study, 46% of individuals met clinical cut-off for autism spectrum condition on the SCQ (Berument et al., 1999), which concurs with previous SAS literature (Zarate et al., 2019, 2021).

The findings also showed convergence across measures (e.g., TAQ impulsivity and ADAMS manic/hyperactive behaviour were associated with aggression) and are in line with previous research identifying impulsivity and overactivity as robust risk markers for aggression in genetic syndrome and intellectual disability populations (Arron et al., 2011; Davies & Oliver, 2016). In this study, comparative behavioural analyses showed that both property destruction and aggression were more likely to be present in individuals with higher levels of compulsive behaviour and general anxiety characteristics. Property destruction was also more likely to be present in individuals with a higher chronological age and males with SAS, while presence of aggression was further associated with higher levels of impulsivity and hyperactivity. Regression models for these behaviours indicated that the variables explaining the most variance were compulsivity and male sex for property destruction, and impulsivity and compulsivity for aggression. It is of note that 55.6% of individuals were reported to show two or more forms of BtC compared to 27.2% of individuals who were reported to show a singular form of BtC. Inspection of the data revealed that property destruction rarely occurred alone. Many individuals who showed property destruction were also reported to show aggression and this behavioural overlap might explain similarities in the correlates associated with these behaviours. The frequent occurrence of aggression and property destruction together in SAS alludes to a build-up of frustration or distress that may lead to BtC presenting within emotional outbursts or temper outbursts (Chung et al., 2022; Woodcock et al., 2011; Tunnicliffe et al., 2014). However, further research is needed to understand the temporal sequences of BtC in SAS, which may indicate related emotional and cognitive processes (Tunnicliffe et al., 2014).

Chronological age has been associated with multiple forms of BtC in previous research in other intellectual disability and genetic syndrome populations, with increased rates of BtC typically seen in older individuals (Crawford et al., 2019; Davies & Oliver 2013; Holden and Gitlesen, 2006). In the current study, presence of property destruction was significantly associated with a higher chronological age. This suggests property destruction may be persistent into adulthood in individuals with SAS. In an intellectual disability sample, aggression and destructive behaviour are reported to be more likely to occur in individuals without motor impairments (Simó-Pinatella et al., 2017). As many individuals with SAS experience health difficulties likely to impact mobility (Zarate et al., 2018, 2019, 2021; Zarate & Fish, 2017), individuals with SAS might be more likely to cause damage to property as they become stronger and more mobile, which may mean destructive behaviour is more noticeable to parents and caregivers as individuals age. The presence of other categories of BtC in SAS may be transient, such as a curved association where a BtC subsides and re-emerges later in life. The cross-sectional design of the current study has limited ability to detect potential age effects. Longitudinal research is therefore needed to delineate the persistence and stability of self-injury, aggression, and property destruction in SAS. This would also enable identification of longitudinal correlates for the persistence of categories of BtC in this group.

In contrast to previous research in intellectual disability and some syndrome groups (Arron et al., 2011; Cooper et al., 2009; McClintock et al., 2003; Murphy et al., 2005; Oliver et al., 2012), adaptive and communicative ability were not associated with presence of BtC in this study. Given impairments in adaptive and communicative ability were evident across all SAS behavioural groups, the lack of association may be explained by a lack of variability in adaptive and communicative ability across the group. Additionally, the use of the Wessex Questionnaire as a proxy measure to estimate the degree of disability may have oversimplified the intellectual and developmental profile of the participants, limiting the ability to detect the potential influence of adaptive ability on the presence of BtC. Similarly, communicative ability was based on the presence or absence of verbal speech according to the Wessex Questionnaire, which does not consider receptive or expressive communicative ability and the use of alternative methods of communication commonly used in SAS (Thomason et al., 2019; Zarate et al., 2021). The Wessex Questionnaire has been used as a proxy measure of disability and communication in previous genetic syndrome research due to it being suitable for large scale questionnaire studies (Bissell et al., 2018; Bozhilova et al., 2023; Kushlick et al., 1973; Laverty et al., 2023; Perry et al., 2022; Waite et al., 2022). Using the Wessex Questionnaire in the current online study helped to maximise the sample size, however, a more detailed assessment, such as the Vineland Adaptive Behavior Scales (Sparrow et al., 2016), may be better equipped to capture potential influences of adaptive ability and communication on the presence of self-injury, aggression, or property destruction.

High levels of repetitive behaviour and indications of compromised impulse control suggest impairments in executive functioning may contribute to the presence of BtC in SAS through compromised ability to regulate behaviour (Oliver & Richards, 2015; Richards et al., 2016). Delayed executive development and difficulties with specific elements of executive function are common in individuals with intellectual disability, with differing profiles of executive function reported across syndrome groups (Perry et al., 2022; Spaniol & Danielsson, 2022; Wilde & Oliver, 2017). Core constructs of executive function include working memory (the ability to hold in mind and manipulate information to complete tasks), inhibition (the ability to suppress behaviour) and cognitive flexibility (the ability to ‘task-switch’ and respond to changing demands and situations) (Miyake & Friedman, 2012). Differences in specific executive function constructs have been shown to underly differing behavioural outcomes in some syndrome groups, e.g., compromised inhibitory control associated with more severe and persistent self-injury and aggression in Fragile X syndrome, and compromised cognitive flexibility contributing to emotional outbursts and aggression in the presence of unexpected or undesired change to routine or plans in Prader-Willi syndrome (Crawford et al., 2019; Woodcock et al., 2009). The behavioural dysregulation model is posited to complement operant learning theory and, given combined presence of high levels of repetitive behaviour and impulsivity in some individuals with SAS, may be important for understanding some forms of BtC (Oliver & Richards, 2015). For example, in an operant learning paradigm, an unmet need might act as a motivating operation and lead to BtC, which might be inadvertently reinforced and increase the likelihood of BtC occurring as the behaviour may become a learned response to a given situation overtime. An individual with compromised executive functioning and behavioural regulation may have compromised ability to inhibit learned behavioural responses and stop behaviour once it has started, and might therefore show more frequent or severe BtC, such as self-injury or aggression (Davies & Oliver, 2016; Oliver & Richards, 2015).

General anxiety characteristics were associated with all categories of BtC in the current study. Previous research has described how anxiety can manifest behaviourally in the form of BtC (Oliver et al., 2020; Tarver et al., 2021, Edwards et al., 2022b). Anxiety might act as an establishing operation for the occurrence of BtC in SAS. For example, anxiety may lead to aggression that serves to escape an anxiety provoking situation, subsequently reducing anxiety. Although anxiety characteristics were associated with all categories of BtC in the current study, more evidence of these associations is needed due to the limited validity of the ADAMS in minimally speaking and/or severe intellectual disability populations (Esbensen et al., 2003). However, there is currently a lack of existing measures that are appropriate for assessing anxiety such populations (Flynn et al., 2017). Furthermore, the ADAMS is very broad, for example, several general anxiety items are also non-verbal indicators of pain (e.g., motor tension), making it difficult to determine whether high ADAMS subscale scores reflect anxiety, pain, or other forms of distress.

Despite a large proportion of the variance in presence of each category of BtC being explained by regression models in the current study, there may be other unmeasured characteristics and factors implicated in the presence and severity of self-injury, aggression and/or property destruction in SAS, such as frequently reported sleep difficulties (Cotton et al., 2020; Zarate et al., 2018). In addition, the current study did not examine the role of wider contextual factors, such as behavioural antecedents and motivating operations for behaviour. Further research is needed to better understand how BtC may develop in an operant conceptualisation in SAS. Sleep difficulties and the presence of painful health difficulties or dental problems are examples of potential setting events that may increase the likelihood of BtC in situations typically associated with these behaviours, e.g., at times of high cognitive demand (Carr & Blakeley-Smith, 2006; Carr & Owen-DeSchryver, 2007; Cotton et al., 2020; Zarate et al., 2018). Environmental contingencies such as social attention may also influence behaviour. Similarly to the strong motivation for social contact seen in Smith-Magenis syndrome (Taylor & Oliver, 2008; Wilde et al., 2013), anecdotal reports indicate strong motivation for social contact from primary caregivers in individuals with SAS. Therefore, lapses in social contact may lead to attention-maintained episodes of BtC in this syndrome group. Future research should examine behavioural antecedents and motivating operations which may lead to maintaining factors for BtC in this syndrome group.

### Limitations

This study has several limitations. Several of the measures required parents/caregivers’ inference about the internal states of the person they care for (Emerson et al., 2013) as well as requiring parents/caregivers to report on behaviour of the person they care for retrospectively. This may have posed challenges for the accuracy of caregiver responses; however, parent and caregivers are best placed to report on the characteristics of the person they care for due to the level of intellectual disability and verbal ability in SAS. The use of informant-report questionnaires may be vulnerable to subjective response bias and common method variance, which can inflate associations between variables (Shihata et al., 2016). Future research should therefore consider the use of direct observations and assessments with individuals with SAS. Furthermore, the sample were recruited via syndrome support groups, and it has been shown that caregivers are more likely to engage with support groups if they care for a person experiencing greater difficulties (Hyman et al., 2002). Although the current sample represents the largest SAS behavioural dataset to date, this may mean the sample lacks individuals with lower levels of support needs.

Regression analyses are difficult to conduct with rare syndrome groups where participant size for analyses is governed by the rarity of a given syndrome. A strength of the current study is that it included the largest SAS behavioural dataset to date; despite this, analyses conducted may be underpowered with reduced ability to detect small effects. Comparative analyses were conducted to inform a priori decisions on which variables to enter in the models, enabling a reduced number of predictors to be entered in each model to improve power. Furthermore, more conservative p-values were selected due to multiple comparisons. The use of comparative analyses to select the variables for the regressions may have meant some variables that could have become significant in the regressions were missed. Given the focus on a rare and under researched syndrome group, it was considered important to identify factors that may be most important to follow up first in future research in this group. The exploratory nature of the comparative analyses has allowed for these factors to be identified; however, additional validation of these findings is important to build causal models for BtC and inform methods of early behavioural intervention in this group. Such work should consider the use of direct observations and assessments with individuals with SAS. Future research should also explore longitudinal correlates of risk for the persistence of BtC in SAS and consider investigating wider contextual factors, such as behavioural antecedents and motivating operations, that may be implicated in the development and maintenance of BtC in this syndrome group.

## Conclusions

This is the first study to identify potential SAS-associated correlates of risk self-injury, aggression, and property destruction, extending knowledge and understanding of these behaviours in SAS. The current study’s findings indicate clinical value in using person characteristics and behavioural indicators of pain or discomfort to identify individuals with SAS who may be more likely to show different forms of BtC. This raises implications for support of individuals and their families. To reduce the development and maintenance of BtC in this syndrome group, professionals should consider assessment of characteristics that may increase the likelihood of BtC, such as physical health, impulsivity, and socio-communicative differences. Early and ongoing assessment of characteristics would facilitate implementation of appropriate early intervention and support strategies for individuals with SAS in the future, such as encouraging use of alternative communication strategies, that might reduce the occurrence of BtC, and poor long-term outcomes frequently associated with BtC in intellectual disability and genetic syndrome populations.

## Data Availability

The data that support the findings of this study are not publicly available. Due to the sensitive nature of personal data collected, participants were not asked to provide consent for data sharing as part of their research participation.

## Footnotes

1 Additional consideration was given to the use of an independent t-test, given that this test is sensitive to violations to HOV when group sizes are unequal (Erceg-Hurn & Mirosevich (2008). Group sizes were inspected for each comparison; a smaller- to larger group ratio of 1: ≥1.5 was considered unequal (Stevens, 2013). Mann-Whitney U or independent t-tests were conducted for comparisons with equal groups. A three-step approach was adopted for unequal group comparisons: 1) unequal variance (Welch) t-tests were conducted if data met assumptions of normality and HOV, 2) independent t-tests were conducted where data violating assumptions of normality/HOV could be transformed, and 3) Mann-Whitney U tests were conducted where data violating assumptions of normality/HOV could not be transformed.

2 In one regression model (aggression) the predictor ‘RBQ compulsive behaviour’ violated the assumption of linearity and data were transformed using inverse square root transformation for aggression presence (SAS + AGG) and aggression absence (SAS-AGG) groups.
